# An alarming but self-limited case of isolated large spontaneous liver hematoma in pregnancy

**DOI:** 10.11604/pamj.2013.14.36.1829

**Published:** 2013-01-25

**Authors:** Vikal Chandra Shakya, Mohan Chandra Regmi, Pannalal Sah, Sudeep Khaniya, Shailesh Adhikary

**Affiliations:** 1 Department of Surgery, B. P. Koirala Institute of Health Sciences, Dharan, Nepal; 2 Department of Obstetrics and Gynecology, B. P. Koirala Institute of Health Sciences, Dharan, Nepal; 3 Department of Radiology, B. P. Koirala Institute of Health Sciences, Dharan, Nepal

**Keywords:** Liver hematoma, pregnancy, HELLP syndrome

## Abstract

Spontaneous subcapsular liver hematoma is rare but potentially life-threatening complication of pregnancy usually associated with severe preeclampsia and HELLP syndrome (hemolysis, elevated liver enzymes, and low platelets). We present here a case of such a large spontaneous liver hematoma presenting in pregnancy, but without other known associated abnormalities, which has not been described before and it resolved on itself without any intervention.

## Introduction

Spontaneous subcapsular liver hematoma is an extremely rare but potentially life-threatening complication of pregnancy, with a quoted incidence of approximately 1 per 45 000 live births [1]. This entity has usually been associated with severe preeclampsia or with HELLP syndrome (hemolysis, elevated liver enzymes, and low platelets), as more than 80% of cases occur in patients with preeclampsia/eclampsia and/or HELLP syndrome, but that occurring without HELLP syndrome or ecclampsia is unusual [2]. We hereby present a case of spontaneous large liver hematoma presenting in pregnancy, without other associated abnormalities, which has not been described before.

## Patient and observation

**Figure 1:**
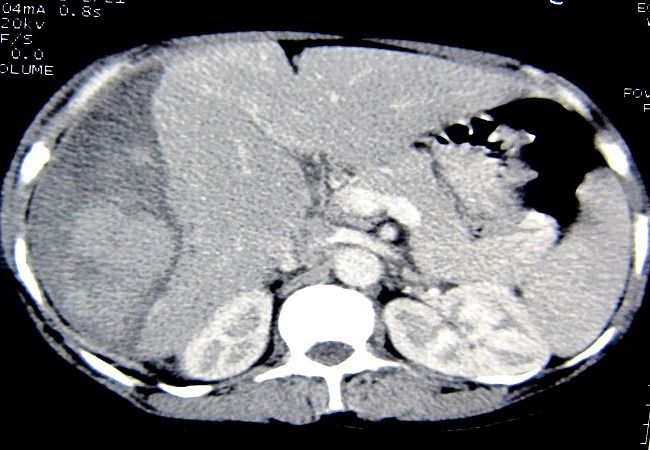
Axial CT scan showing large liver hematoma compressing the liver

**Figure 2:**
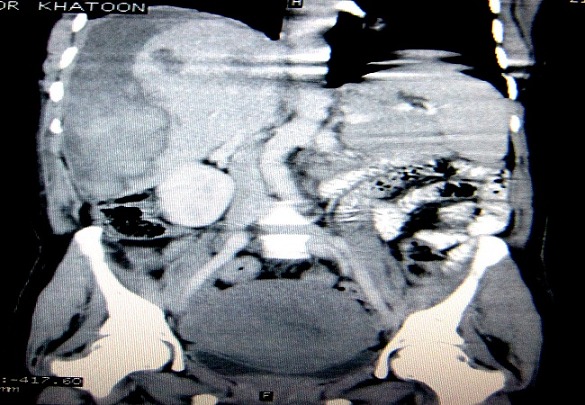
Reconstucted 2-D coronal image shows the large subcapsular liver hematoma and few intraparenchymal hematomas

A 30 year female, gravid 7 para 7, with uncomplicated previous pregnancies, presented to hospital with history of bilateral subcostal pain more on the right side and not perceiving fetal movements for last 5 days at 27 weeks of pregnancy. There was no history of trauma, jaundice, any bleeding disorders previously. On examination, she was found to have mild pallor, and a tender hepatomegaly. There was no icterus and the patient was hemodynamically stable, her blood pressure was also normal. Ultrasonography revealed intrauterine fetal death and a subcapsular hematoma of size 8.9cm X 8.6 cm in the liver. Computed tomography showed a large subcapsular hematoma almost occupying 25% of the liver volume causing compression and indentation of liver parenchyma and few hepatic intraparenchymal hematomas (Figure 1 and Figure 2). Her laboratory parameters were as follows: hemoglobin 10.2gm%, total leucocyte counts 11000/mm^3^, platelets 2,79,000/mm^3^, prothrombin time 13/sec, INR 1.0, bleeding time 3 min, clotting time 7min, peripheral smear showed normocytic normochromic to mildly hypochromic picture with adequate platelets adequate, total protein 5.5 gm%, serum albumin was 2.6 gm%, total bilirubin was 0.9gm%, conjugated bilirubin 0.2 gm%, aspartate aminotransferase 330 IU/li, alanine aminotransferase 270 IU/li, and alkaline phosphatase was 323 IU/li. Her routine and microscopic examination was also normal. She was conservatively managed, as she was stable, with complete bed rest, prophylactic antibiotics and analgesics. She didn't require any blood transfusions. Due to intrauterine fetal death, she underwent an induced abortion on 5^th^admission. Her condition remained static and was discharged after a watchful period of next 5 days. She is asymptomatic during follow-up visits till a period of 6 months, her serial sonographic scans show gradual decrease in the size of the hematoma.

## Discussion

As first described by Abercrombie in 1844 as a complication of pregnancy, spontaneous liver rupture remains a rare event [3]. There have been over 100 cases reported in the literature, with the majority being associated with pregnancy-induced-hypertension as well as primary and metastatic liver tumors [4, 5]. This has also been described in patients with HELLP syndrome, Ehlers Danlos disease and graft-vs-host disease [6–8]. There is an almost exclusive association with severe preeclampsia or with HELLP syndrome (hemolysis, elevated liver enzymes, and low platelets), as more than 80% of cases occur in patients with preeclampsia/eclampsia and/or HELLP syndrome [2]. The clinical presentation of subcapsular hematoma is not characteristic; most patients present with right upper quadrant pain [1]. However, due to the rarity of this entity and its variable presentation, most cases are missed and diagnosed only at laparotomy.

The peculiarity of the present case is that, the lady was normotensive, and there were no other associated bleeding disorders or significant biochemical abnormalities. Subclinical trauma could have been a possibility to put as a cause. Most of the literature searches show that liver hematomas are associated with ecclampsia; the hematoma ruptures, and the patient presents in shock. We believe the slight elevation in liver enzymes is due to compression of the liver by the large hematoma rather than the cause of the hematoma. The pathogenesis of subcapsular liver hematoma and subsequent rupture is unclear. Fibrin deposition in the hepatic sinusoids is speculated to be the initiating event [9]. Fibrin deposition may lead to platelet activation, thrombus formation, occlusion of capillaries, and subsequent hepatic hemorrhage and necrosis. Coalescence of these hemorrhagic areas leads to dissection of the Glisson capsule from the liver surface. Concurrent consumptive coagulopathy occurring in preeclamptic patients often aggravates the condition. A tense subcapsular hematoma may rupture spontaneously or secondary to trivial trauma during labor or convulsions, leading to catastrophic life-threatening hemorrhage. Awareness of this entity and its early recognition is important in reducing the morbidity and mortality associated with a ruptured liver hematoma.

## Conclusion

Liver hematomas can occur in a pregnancy without other associated abnormalities and found to resolve by itself. Though looking alarming, awareness of this entity and visualization with computed tomography can help to explain the prognosis to the patients
